# Acquired Idiosyncrasy for Quinine, Showing Peculiar Cutaneous Manifestations*Read before the American Dermatological Association at Its Eighteenth Annual Meeting.

**Published:** 1895-02

**Authors:** Charles W. Allen

**Affiliations:** New York, Surgeon to the City Hospital, Genito-Urinary Division; Visiting Dermatologist to the Randall’s Island Hospital, Etc.


### ACQUIRED IDIOSYNCRASY FOR QUININE, SHOWING PECULIAR CUTANEOUS MANIFESTATIONS.*

BY CHARLES W. ALLEN, M. D., NEW YORK,

Surgeon to the City Hospital, Genito-Urinary Division; Visiting Dermatologist to the Randall's Island Hospital, Etc.

[*From Medical Record.*]

The skin eruptions which follow the use of the cinchona barks and their derivatives possess a double importance from the wide-spread employment of these drugs and their consequent liability to occasion frequent rashes, and the fact that, although the literature of the past ten years, at least, is not lacking in reports of cases, many of them show that the drug had either been persisted in for some time after the skin manifestations have appeared, through ignorance of their nature and cause, or else that it has been administered for a second time, and then only when the same eruption showed itself again has its origin been suspected.

It is for this reason that I venture to consume a little of our time in the report of a case possessing, it seems to me, some features of interest; and I hope the discussion will aid in the elucidation of some points which we must admit are unsettled, concerning the action of the drug in producing those phenomena with which we are familiar.

All of us have seen instances of pruritus due to quinine in which no exanthem shows upon the surface. I can recall a number of cases in which this tendency manifested itself in more than one member of a family; thus, a father and daughter, a mother and son, will have the same peculiarity, and I have repeatedly had patients tell me they could not take quinine, nor could one or the other parent before them.

The most frequent form of eruption resulting from quininism is probably the scarlatinoid, an erythematous rash, beginning usually upon the face, and subsequently extending over the whole surface. Other usual forms are the urticarial and papular, while the vesicular, bullous, and purpuric are less frequently encountered; and last of all come the gangrenous forms. Dr. Van Harlingen, in his interesting and instructive paper on "Medicinal Eruptions," read before this Association

*Read before the American Dermatological Association at its Eighteenth Annual Meeting.

in 1880, says that in exceptional cases the eruption may not become generalized, and Dr. Morrow uses the same words in his well-known monograph, in which is incorporated a paper written by himself, also in 1880; both agreeing that the erythematous is the prevailing type of eruption. Dr. Morrow found thirty-eight out of the sixty cases analyzed to belong to this variety.

The case I now report differs from the majority of those found in literature: (1) In that the eruption occurred in a subject who had previously always been able to take quinine in moderately large doses (gr. v. to gr. xv.), and had never before suffered in any way from its use. (Dr. Morrow refers to this acquired idiosyncrasy in a case of his own.) (2) In that the eruption is developed by a smaller quantity of the drug than I have been able to find recorded in any other previous observation. (3) In that the lesions are local and show no tendency to spread or to become generalized. (4) In that the location of individual spots is always the same, no matter what the dose. (5) In that the eruption was produced four times accidentally, and some fourteen times experimentally. (6) And finally, in the fact that varying with the dose, and possibly with other unknown conditions, the lesion, which is always primarily, and usually solely erythematous, may become urticarial, œdematous, bullous, covered over with small vesicles, or converted into an excoriated patch. In other words, the simple rosy erythema of one attack may closely simulate in another an erythema exudativum multiforme. The amount of irritation in one case being, it would seem, sufficient to produce only a dilatation of the vessels and redness of the skin, while in another an exudation takes place which may either show itself as an œdema, or if fluid is poured out between the corium and epidermis, the latter may be raised into a bullous sac, or numerous small vesicles may spring up to stud the surface. A greater intensity of action, or the same degree exerted upon delicate parts, such as the prepuce in this instance, may cause rapid shedding of the epidermic layer, and leave exposed a tender, excoriated, and bleeding area.

In 1889, the subject of this sketch, while suffering from toothache, took a five-grain dose of the sulphate of quinine at bed time. During the night there was decided general pruritus, and in the morning large, round erythematous blotches were found upon the backs of the hands, inner surfaces of the thighs above the knees, dorsum of the foot, glans penis and prepuce, as shown in the drawing on page 99. With the exception of a blotch on either side of the spinal column, opposite the third or fourth vertebra, the size of a fifty and a twenty-five cent piece, respectively, these were the only regions in which spots appeared in this or any subsequent attack. Having suffered from urticaria on two previous occasions from eating clams, the idea of this eruption being of the same nature was suggested to the man's mind, and not suspecting the drug, which he had often taken before in larger quantities, the dose was repeated. The symptoms became more pronounced, and by the next morning, when I saw the eruption, the prepuce presented two large, oval excoriated surfaces, which became in a few days so suggestive of large chancroids that a colleague, to whom they were shown, suspected at first that this was really their nature. The rest of the region of the glans and prepuce was intensely red and sensitive. From the history (for I had never before seen just such an eruption from the drug) I made the diagnosis of exanthem due to quinine, and ordered its use discontinued. In about a week the spots began to desquamate, and continued to fade in color while peeling for about ten days longer. On July 2, 1890, again suffering from neuralgic pain in the teeth, the patient took one "Mousette" pill, not knowing or not remembering that it contained quinine (about one grain). Within twelve hours itching, burning, and prickling sensations began, and the same spots as in the first instance showed themselves. Two days later the whole prepuce was excoriated as after a blister, and there were digestive disturbances. On July 6th, the skin over the first phalanx of the little finger on the right hand was tender and looked like a burn. On the 9th, the glans penis would bleed when irritated, while the prepuce was in a healing condition. By the 12th, desquamation was progressing in the various blotches. Again, in this same year, a cordial glassful of "Bougeaud" coca wine was taken and the same condition followed, though in a less pronounced way. The quantity of the drug in this instance must have been exceedingly small.

On August 18, 1891, patient took, in a drug store, a little elixir of calisaya with Vichy, the equivalent of quinine not exceeding one-third grain. Half an hour later, well-recognized tinglings brought a realizing sense of the inadvertence. Within a few hours the spots had appeared, and the glans penis was encircled as by a ring of redness. There was no elevation of temperature. Two days later there was a bulla upon the little finger raised one-quarter of an inch above the niveau. Over some of the other spots were scattered miliary vesicles, and some spots showed enclosed rings resembling erythema iris. The bulla was pricked, and the serum allowed to flow off. The wall dried down into a waxed-paper-like scale. The skin beneath this was tender, and desquamation was repeated several times before the redness disappeared. Glans penis and prepuce became denuded of epithelium, and glans desquamated after healing had taken place. Thus it is seen that four times an eruption very similar in kind and degree followed the ingestion of quinine in varying quantity. Three times it may be said to have been accidentally taken after the first had demonstrated the idiosyncrasy.

In order, if possible, to clear up some of the things which are still unsettled concerning quinine eruptions, I now undertook a series of experiments with the co-operation of so favorable and, as he proved, willing a subject.

The statement has been made and repeated that it is the acid, and not the base, which is the offending substance in these cases, and that the individual who breaks out after the sulphate can take the citrate, for example, with impunity. To determine this point, at least so far as this case was concerned, and at the same time to test the efficacy of the tannate of quinine, which has been accused of being an inert preparation, I gave one chocolate tablet containing one grain of tannate, such as are prepared for children.

In exactly two hours a decided pruritus, tingling and burning, began to be felt in the region of the prepuce. This was found on inspection to be of a bright scarlet, as was also the glans penis. Stinging points were noted in the habitual regions, especially in the interscapular area. Scarlet blotches followed shortly in all of the usual situations. During the following day some of the hyperæmic patches became noticeably raised above the surface and somewhat œdematous, pitting upon pressure, while others were tender to the touch, and, as noted before, felt and looked like burned spots.

Allowing abundant time for the full effect of each exhibition of the drug to wear off, I tried in succession the hydrobromate or bromide of quinine in dose of one-quarter of a grain; the hydrochlorate, the bisulphate, dextro-quinine, and the bimuriate with urea (a soluble product for hypodermic use going under the name of quinine carbamide), all in the same dose. These were invariably followed by the same manifestations I have described, and when taken in connection with the recorded instances of eruption from the various other preparations, including the citrate, I believe the claim of Giampetro and others, that the acid and not the base is at fault, cannot stand.

Our next attempt was to find out whether the drug might not, by circulating through the lymphatics, be brought into contact with certain nerves of the skin or terminal nerve filaments, and produce in them a local effect. To test this a tight band of adhesive plaster was bound about the wrist of the right hand so as to make decided pressure, and a dose of hydrochlorate of quinine was administered by the mouth. Despite this constriction, kept up for the whole of an afternoon, the spots appeared upon both hands as usual, and a little earlier, if anything, upon the right than upon the left. This experiment was repeated a month later by applying the constriction to the forearm, and after the hand was cyanotic, injecting into the skin and subcutaneous tissues, between the hand and the band, one-quarter grain of the carbamide in solution. The effect of this was to produce after ten minutes an anæsthetic area the size of a fifty-cent piece, with slight and transient œdema, leaving behind an infiltrated condition of the skin with numbness of the whole back of the hand. For two days the site of injection was tender and the arm had a slightly lame feeling, but no erythema or eruptive signs of any kind appeared at the site of injection. The injection was made at noon, and at 12:20 the itching began to be felt in the prepuce. At 12:45 there was a slight earache, a symptom noticed once or twice before shortly after a dose had been taken. At 5 P. M. the erythema began to appear on the prepuce and right hand, and at 8 P. M. on the left, followed by spots in the other usual locations.

On another occasion $\frac{1}{100}$ grain of the hydrochlorate was injected into the skin of the right arm, and an equal quantity into the hand at the site of one of the erythematous blotches, but aside from what might be expected from the injection of the same amount of water, there was no effect. I therefore came to the conclusion from these trials that the drug was incapable of acting in a local way, and that its action could not be confined to a given region, or prevented from entering this region by mechanical compression of the superficial vessels and lymphatics. They also showed that the theory of reflex dilatation of the vessels in the skin from stimulation of the sensory nerves of the stomach was not applicable here, unless we presume that enough of the drug is carried by the circulation to the gastric mucous membranes, when injected beneath or into the skin. But why should we presume that there would be primary irritation of the stomach nerves, if we are unable to produce this effect upon the skin nerves?

To test the effect upon mucous surfaces, one-quarter grain of the carbamide in solution was held by the patient in his mouth for fifteen minutes. No local effect of any kind was noted, and no general effect for six hours, when erythematous spots appeared in their old locations near the left knee, on the dorsum of the left foot, and upon the right little finger. These spots faded after two days and no others appeared. After some days the same quantity of the same preparation was made into a suppository with cocoa butter, and introduced just within the sphincter ani, where it was allowed to melt. No local effect whatever, but the general symptoms followed in quite a pronounced way after five hours, and the spots did not entirely fade for two weeks. If we consider the effects in these instances as reflexes from irritation of the digestive tract, should we not expect a rather quicker result than when we inject the drug into the skin? Such was, however, not found to be the case. To test the possibility of local effect from inunction, I had prepared an ointment containing two grains of the hydrochlorate in a small quantity of lanoline and lard. This was rubbed into the back of the patient's right hand for fifteen or twenty minutes. No local effect was appreciable, but about six hours later a distinct erythema of the corona glandis and prepuce appeared, with the attending pruritus, and lasted twenty-four hours. There was also irritability about the neck of the bladder, with frequent calls to urinate, and two days later a herpes præputialis developed, causing further trials to be postponed. The herpes was not looked upon as a direct effect of the quinine, but it was thought that it might be referred indirectly to it through the bladder symptoms. Patient stated that in former years he had been troubled with frequent attacks of herpes, but for six or eight years did not remember to have had it.

Although I am quite well aware that quinine in susceptible subjects often causes vesical irritation, I did not lay great stress upon this instance, from the fact that this patient had often suffered in a similar way when he "catches cold," and when no quinine had been taken; and, on the other hand, in most of the eruptive attacks the bladder function had not been interfered with. Some time after the herpes had healed, four grains of the muriate, in ointment, was again rubbed into the hands for twenty minutes at bed-time. In the early morning there was thickening of the skin of the prepuce, redness extending over the whole glans, irritability of the bladder, and frequent urination, continuing for thirty-six to forty-eight hours, also slight sciatica-like pains in the right leg. The effect upon the hands was slight, the little finger being more affected than other parts, desquamation taking place several times in succession. There was no evidence that any local action had resulted from the inunction. I now became convinced that the vesical irritability did proceed from the quinine absorbed by the skin. Cases in literature show that this effect is recognized, and that hæmaturia even may be occasioned, just as it may be cured, by quinine. This may be homœopathic in principle, but the dose necessary for this result is usually far from being one of homœopathic proportions. Still, with the view of testing skin absorption, I had the man apply to the scalp with moderate friction a drachm of an alcoholic solution containing one grain of quinine. Twelve hours later there was erythema of the glans and upper surface of the prepuce, with decided tingling and burning, but no local change on scalp which could be charged to the drug. Subsequent daily applications of half this quantity did not seem to increase the intensity of the original effect.

This completes my observations in the case, but I have purposely omitted till now the mention of a symptom to which I am inclined to attribute some importance in determining the manner in which quinine may act in the production of cutaneous eruptions.

In the first and second attacks, in that brought on by the hydrobromate three years later, and in the last outbreak after rectal administration of the drug, complaint was made of a dull ache in the interscapular region. This was more pronounced in the early attacks. Examination on repeated occasions showed the undoubted existence of marked tenderness on pressure over the spinous process of the second dorsal vertebra. This tenderness would disappear as the eruption faded, and was never to be fonnd in the intervals of quinine eruption.

Now, upon this symptom, indefinite as it may be, I pivot my proposition, or, may I say better, supposition, that the eruption is due neither to a pathological reflex direct from the digestive tract; nor to a direct effect upon the peripheral nerves; nor to the irritation produced locally upon the skin in the process of elimination of the drug by the glands; nor to its action as a protoplasmic poison upon the capillary vessels; but, on the contrary, to an angeio-paralysis of central spinal origin. Whether the drug undergoes chemical change before it is capable of producing these effects, I am unable to say, but from the rapid onset of pruritus I would argue that it acted in its unchanged state.

It is well-known that quinine is eliminated by the sweat-glands as well as by the kidneys, but the constant symmetrical distribution, and circumscribed features of the eruption in this case, it seems to me, are much more rationally explained on the theory of nerve origin.

Again, in support of this we have an effect prolonged much beyond what one would expect from the irritation due to elimination. We know that quinine can be detected in the urine within ten minutes of its hypodermic injection, and that it may be found here for several days; but presuming the elimination by way of the skin to be likewise protracted, it would scarcely account for instances in which an active stage of eruption or apparent exacerbations continue for several weeks after the drug is withdrawn. If we invoke the direct local action of the drug upon the bladder-wall, for instance, to account for vesical irritation, we must presume that the drug has undergone some change in the system, since simple contact with mucous membranes, as I have shown, produces no irritation or local effect.

I cannot agree with Bouvard that skin elimination explains all the eruptions, as he claims, excepting that of purpura, which may have its source in the action of the drug upon the blood and tissues.

That hyperæmia of certain regions follows a moderately full dose is shown, for example, by observing the ear-drum or the fundus of the eye; the fullness of the vessels indicating a corresponding intra-cranial effect. That the cerebral circulation is quickened is shown by the exhilaration often secured by a comparatively small dose. Thus I have as a patient a reverend gentleman who is in the habit of taking two or three grains of quinine upon starting for church. The beautiful choice of words and eloquence of the sermon make the ears of his hearers fairly tingle, and not one of his congregation dreams that their tinnitus is produced by the preacher's cinchonized words.

Full doses, too, as is well-known, will cause dilatation of the pupil similar to that from belladonna. These effects may all originate in the nerve-irritation. In large doses, Chapron says, the reflex function of the cord is lessened and ultimately abolished; and Wood has pointed out that convulsive attacks may follow.

If large doses can produce such marked results by acting upon the cord, why is it not rational to refer these skin changes to the same source?

The analogy which certain of these quinine eruptions bear to urticaria and circumscribed œdema, depending probably on innervation disturbances, must not be overlooked, especially the angeio-neurotic œdema that occurs in subjects of urticaria who are distinctly neurotic or give history of neuralgic attacks, etc. The vaso-motor disturbance here, as well as in certain quinine eruptions, is probably first spasmodic and secondly paralytic. I would also mention the somewhat analogous condition of symmetrical œdema and other trophic changes in the extremities, in those states in which the cord is known to be the seat of pathological change—I refer to syringomyelia, Morvan's disease, Raynaud's disease, etc.

The only case I have been able to find similar to my own was reported by Dr. Munn in the *Atlantic Medical and Surgical Journal*, in 1879. His patient always developed bright-red erythematous patches upon the left cheek, nose and chin, the palmar aspect of the left wrist, the back of the left hand over the carpo-phalangeal articulations of the fourth and fifth fingers and the left knee. In his case, however, doses ranging from five to thirty grains were required, and the eruption only developed after the drug had produced its full effect on the system. The tendency ceased after an attack of pneumonia and did not return, thus showing that the acquired idiosyncrasy may fortunately be lost again.

I do not wish to enter into the matter of treatment, excepting to draw attention to what might be a misleading statement under this head in Morrow's "Drug Eruptions." The author says, "Treatment is hardly ever necessary for the cutaneous disorders caused by quinine, since they spontaneously disappear on withdrawal of the offending agent." This might lead to the supposition that the rash would be gone the day after the quinine was stopped, whereas several weeks may elapse before the activity of the eruption and pruritus have ceased entirely. Indeed, the eruption may not even appear until after the drug has been withdrawn for some time. I have also been unable to verify the statement that women are more susceptible than men to quinine eruptions. My personal experience has led to the opinion that sex plays no part in their production, and that the cases are about equally distributed.

If there is anything in this theory of spinal irritation, it may have an importance not only as bearing upon the etiology of certain obscure skin affections, but also as offering a possible explanation of the way quinine acts in producing some of its therapeutic effects.

